# The Mitochondrial Lon Protease: Novel Functions off the Beaten Track?

**DOI:** 10.3390/biom10020253

**Published:** 2020-02-07

**Authors:** Wolfgang Voos, Karen Pollecker

**Affiliations:** Institute for Biochemistry and Molecular Biology (IBMB), University of Bonn, Nussallee 11, D-53115 Bonn, Germany; karen.pollecker@uni-bonn.de

**Keywords:** mitochondria, Lon protease, protein quality control

## Abstract

To maintain organellar function, mitochondria contain an elaborate endogenous protein quality control system. As one of the two soluble energy-dependent proteolytic enzymes in the matrix compartment, the protease Lon is a major component of this system, responsible for the degradation of misfolded proteins, in particular under oxidative stress conditions. Lon defects have been shown to negatively affect energy production by oxidative phosphorylation but also mitochondrial gene expression. In this review, recent studies on the role of Lon in mammalian cells, in particular on its protective action under diverse stress conditions and its relationship to important human diseases are summarized and commented.

## 1. Introduction

Proteins of the AAA+ (ATPases associated with various cellular activities) family belong to large and diverse group of enzymes characterized by the formation of oligomeric (mostly hexameric) ring structures. The mechanistic basis of the function of many family members seems to be the threading of linear macromolecules, mainly polypeptides but also DNA, through the central pore driven by ATP-dependent conformational changes in the AAA domains [1]. These structural and mechanistic properties have also been utilized by a specific family of proteolytic enzymes that are responsible for the degradation of intracellular polypeptides [2]. A specific feature of these proteases is that their active sites are shielded from the environment inside a large oligomeric complex. This protein complex forms an internal degradation chamber so that only polypeptides can be degraded that are inserted into this chamber in an active and regulated process. A typical example for such “chambered proteases” is the proteasome in the cytosol of eukaryotic cells [3]. Somewhat simpler versions of chambered proteases exist in all domains of life, in bacteria, plants, fungi and metazoan organisms. An important feature of chambered proteases is that they always contain a proteolytic activity and a chaperone-like activity that are structurally separated, either as separate domains on a single amino acid chain, or as two separate polypeptides forming a tight cooperative protein complex. Typically, the chaperone component contains the AAA domain that is responsible for substrate recognition, remodeling of its structure and insertion in the proteolytic chamber. This specific mechanistic and/or functional cooperation between proteases and molecular chaperones represents the main hallmark of cellular protein quality control (PQC) systems that are required to maintain protein function under normal and stress conditions, a process summarized under the expression “protein homeostasis” or “proteostasis”.

As endosymbiotic organelles, the overall properties of the mitochondrial PQC system are very similar to those found in their bacterial ancestors. Therefore, inner membrane and matrix compartments contain proteolytic systems highly similar to bacteria (in part even carrying the same names): the soluble proteases Lon and ClpP and the membrane-integrated m-AAA and i-AAA protease complexes. These four proteolytic enzymes contain typical AAA-domains and form chambered protease complexes responsible for general protein degradation with broad specificity. As a rule-of-thumb, the two soluble proteases are mainly degrading substrate proteins in the matrix, whereas the inner membrane AAA proteases degrade membrane-integrated targets. The m-AAA protease active site faces the matrix compartment, whereas the i-AAA site the intermembrane space. Another broad-specificity protease, HtrA2 (high temperature requirement protein A2), exists in the intermembrane space, which employs a different mechanism for protein degradation and does not belong to the classical chambered proteases. Note that mitochondria contain many more proteases and peptidases [4]. However, they do not belong to the AAA+ family and also have no major role in PQC.

**Figure 1:**
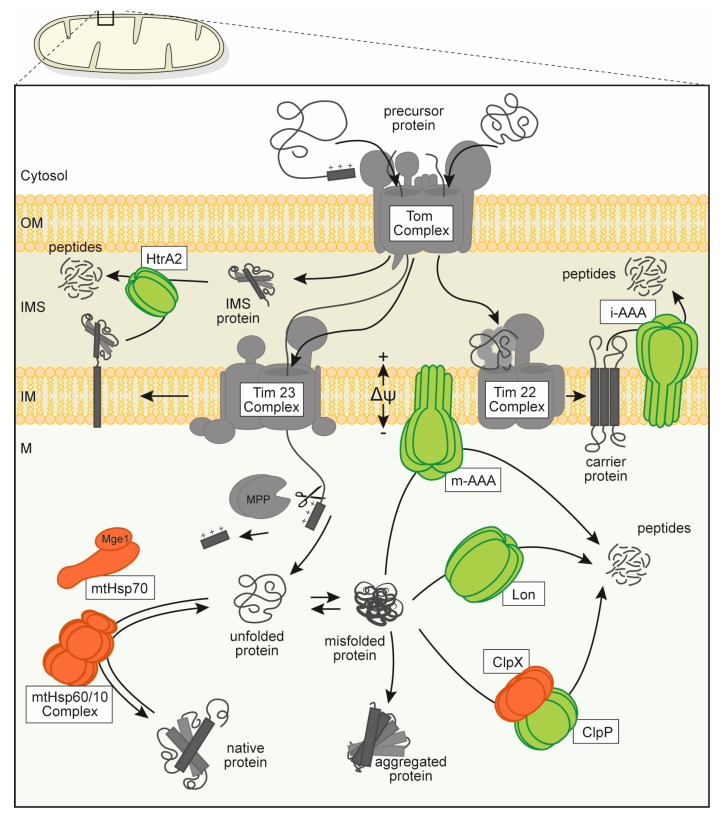
Overview over the mitochondrial protein quality control (PQC) system consisting of several types of molecular chaperones (orange) and oligomeric, chambered proteases (green). Proteins are depicted in a schematic form (not to scale) that is roughly based on available structural data. Mitochondrial protein biogenesis relies on the import of cytosolically synthesized preproteins through the mitochondrial outer and inner membranes via dedicated translocation pores (gray). Proteins translocated into the matrix compartment are usually processed to the mature form and require folding and assembly to their active conformation under assistance of molecular chaperones. Misfolded or denatured polypeptides are also recognized and stabilized by interaction with chaperone enzymes but may be degraded to avoid accumulation of protein aggregates. Abbreviations: mitochondrial membrane potential (Δψ), outer membrane (OM), intermembrane space (IMS), inner membrane (IM) and mitochondrial matrix (M), translocase of the inner membrane, mitochondrial (Tim complex), translocase of the outer membrane, mitochondrial (Tom complex), mitochondrial-processing peptidase (MPP), high temperature requirement protein A2 (HtrA2), intermembrane space AAA protease (i-AAA), matrix AAA protease (m-AAA), genome maintenance exonuclease 1, mitochondrial (Mge1), heat shock protein 70 kDa, mitochondrial (mtHSP70), complex of heat shock protein 60 kDa and 10 kDa, mitochondrial (mtHsp60/10 complex), ATP-dependent Clp protease ATP-binding subunit clpx-like, mitochondrial (ClpX), ATP-dependent Clp protease proteolytic subunit, mitochondrial (ClpP).

The AAA+ proteases are embedded into a functional network inside mitochondria that is responsible for organellar proteostasis (Figure 1). Apart from the protease enzymes, mitochondria contain the classical chaperone systems known from bacterial cells, in mammals with the core components mtHsp60 (mitochondrial heat shock protein 60 kDa, encoded by *HSPD1*) and mtHsp70 (mitochondrial heat shock protein 70 kDa, encoded by *HSPA9*; also named mortalin or Grp75). These molecular chaperones are responsible for most of the protein folding reactions in the matrix as well as for the stabilization of polypeptides under stress conditions. MtHsp70 is also prominently involved in the translocation reaction of cytosolic preproteins into the matrix compartment [5]. There is some variability in the chaperone content between mitochondria in fungal, plant, and mammalian species, the latter not containing a ClpB-type Hsp100 chaperone, which is mainly responsible to achieve tolerance against elevated temperatures [6]. Mitochondrial chaperones together with the chaperone-like activities of the mitochondrial proteases ensure folding and assembly reactions of newly imported preproteins as well as damage control to prevent accumulation of misfolded polypeptides and protein aggregation, representing a first line of defense against proteotoxic stress conditions [7]. In the recent years, it became apparent that cellular PQC systems show a certain adaptability to stress conditions posed by an accumulation of unfolded polypeptides. Here, an upregulation of PQC gene expression serves to counteract an accumulation of damaged polypeptides even in the absence of heat stress. This so-called unfolded protein response (UPR) has been characterized in detail for ER chaperones. Although indications for a similar process exist also for mitochondria (“mtUPR”), the details and mechanisms, in particular in mammals, are still not entirely clear [8]. In this context, it has to be taken into account that mitochondria may not only have an efficient PQC system, but also underlie a specific organellar quality control (OQC) that acts on a cellular level [7]. In case of a more severe or pathological deactivation of mitochondrial functions, it has been shown that the damaged mitochondria can be removed by a specific variant of autophagy, described as stress-related mitophagy [9]. Importantly, in case of a terminal and non-repairable damage of mitochondria, cells will undergo apoptosis, essentially removing themselves from the organism [10].

The protease Lon plays a central role in mitochondrial PQC. First, characterized as a soluble ATP-dependent proteolytic activity the mitochondrial matrix [11], the enzyme itself had been identified ~25 years ago in human [12] and yeast cells [13,14]. In the yeast *Saccharomyces cerevisiae*, the Lon protease is encoded by the gene *PIM1* (proteolysis in mitochondria). There is high sequence conservation between the mitochondrial and the bacterial Lon proteases (~37% amino acid identity between the human and *E. coli* protein) and it is generally assumed that they share a similar basic enzymatic mechanism [15]. The human mitochondrial Lon protease is encoded in the nuclear genome by the gene *LONP1*. Note that mammalian cells also contain a second Lon-like protease localized in peroxisomes, encoded by the gene *LONP2* [16]. During cytosolic translation of the *LONP1* mRNA, a 106 kDa large precursor polypeptide is generated that contains an N-terminal mitochondrial targeting sequence. The precursor protein is imported via the standard translocation pathway [17] into the mitochondrial matrix and processed by removal of the presequence to a 100 kDa form. As mitochondrial preproteins pass the membrane channels in a completely unfolded conformation, the processed Lon polypeptide has to fold and assemble into the active enzyme complex in the matrix compartment. The Lon protease consists of three distinct protein domains: an N-terminal domain, responsible for substrate binding, followed by the AAA+ module containing typical Walker A/B ATP binding and hydrolysis sites, and the C-terminal protease domain with a serine at the active site [18]. The structural arrangement of the Lon polypeptides in the active enzyme complex is not completely clarified to date. Based on the limited structural data available so far, the basic form of Lon is represented as a hexameric chambered protease complex, similar to other family members. As the yeast Pim1 exhibited a heptameric open ring structure in EM pictures [19], a substantial asymmetry and/or structural variability, also depending on the functional state of the enzyme, has to be taken into account.

Essentially, the protease Lon performs three major tasks: (i) performing protein quality control in the matrix compartment, (ii) support of mitochondrial gene expression, and (iii) mediating organellar stress reactivity under pathological conditions. Taken together, these molecular functions place the Lon protease into the central hub of the mitochondrial proteostasis network that is required to reach and maintain full mitochondrial functionality. Due to this considerable functional variability and the multitude of mitochondrial processes for which a participation of Lon has been described, it often remains difficult to distinguish a direct functional involvement from accumulative indirect effects. The main purpose of this review is to summarize and comment on recent developments in the functional characterization of the Lon protease, in particular on results that have been obtained studying human cells.

## 2. Lon Function in Mitochondrial PQC

The primary functional characterization of mitochondrial Lon-type proteases was performed initially in yeast cells due to the ease to generate genetic deletion mutants. However, the proteolytic system in fungal mitochondria deviates from the situation in metazoan and plant organisms as fungi lack a homolog of the ClpP protease, and therefore contain only one soluble protease system in the matrix compartment. Yeast deletion mutant cells (*pim1∆*) were respiratory deficient, indicating an important function of the matrix protease Pim1 in maintaining mitochondrial energy metabolism [13,14]. In humans, a genetic disorder—the CODAS syndrome (cerebral, ocular, dental, auricular, skeletal), a multi-system developmental disorder—has been associated with mutations of the *LONP1* gene. Here, point mutations cluster in the AAA domain near the ATP-binding pocket, resulting in a reduction of Lon activity. Similar to yeast cells, mammalian cell models of this disease exhibited the typical phenotypes of mitochondrial PQC deficiencies like morphology defects and reduced respiration efficiency [20]. These disease model cells also showed an aggregation of an mitochondrially encoded subunit of the cytochrome c oxidase complex, MT-CO2, indicating that the observed functional defects correlate with a loss of mitochondrial PQC capacity. Note that despite the obvious phenotype of Lon/Pim1 deletion mutants, mechanistic details of the involvement of Lon in mitochondrial respiration remain unclear to date as its function as protease has no direct biochemical connection to the activity of the enzymes of mitochondrial oxidative phosphorylation. This general negative effect of Lon defects on mitochondrial functions is likely rather indirect in most cases (see following discussion).

### 2.1. Proteolysis Properties

A direct demonstration of the proteolytic function of the Lon protease was obtained by experiments with isolated intact yeast mitochondria where newly imported artificial reporter proteins were degraded in a Pim1-dependent reaction [21]. In this context, a close functional cooperation between the single soluble protease in the matrix compartment with the mtHsp70 chaperone system was observed. Essentially, mtHsp70 would interact with and keep newly imported degradation-prone polypeptides in an unfolded or a at least soluble state. These chaperone-bound misfolded polypeptides then would serve as direct substrates for Lon-mediated proteolysis. Utilizing an extended set of reporter proteins with varying properties, it was shown that Pim1 requires a short unstructured polypeptide segment of approximately 50–80 amino acids to bind and degrade substrate proteins. This degradation reaction is processive, proceeding from the unstructured segment to the other parts of the substrate polypeptide. However, Pim1 exhibited—similar to other chambered proteases—only a low intrinsic unfolding capacity and was not able to degrade stably folded protein domains. Taken together, these recognition and degradation properties restrict proteolysis by Pim1 to destabilized polypeptides [22]. A similar substrate selection process was also demonstrated for the mammalian Lon protease. In addition, it was recognized that Lon is also capable of cleaving substrate proteins at internal sites as long as they present longer unstructured amino acid segments [23].

A proteomic analysis of the degradation processed of endogenous mitochondrial proteins demonstrated that Pim1 is the main protease responsible for removal of damaged or nonnative proteins in the matrix compartment [24]. Pim1 substrates were mainly proteins with complex structural composition, including oligomeric proteins and enzymes with prosthetic groups or Fe/S centers. Many of these proteins are meta-stable and become susceptible for degradation already under mild stress conditions. The high susceptibility of Fe/S cluster containing mitochondrial enzymes might be even a factor in the regulation of cluster biogenesis. In yeast cells, the scaffold protein Isa, required for Fe/S cluster biosynthesis, is also degraded by the Pim1 protease depending on the cellular demand for the cluster molecules [25,26]. The specific substrate recognition properties of Lon/Pim1 restrict its degradation activity on structurally meta-stable substrate proteins without the requirement for a specific tagging system, like in case of a degradation by the ubiquitin/proteasome system in the cytosol. Although most basic information on the proteolysis properties of Lon was obtained in the yeast system, it was shown that human Lon also exhibits practically identical properties, essentially protecting mitochondria against aggregation by degrading imported destabilized polypeptides [27]. 

As complete deletions of the Lon gene in metazoan organisms turned out to be embryonically lethal [28], most studies on Lon mutants have been performed utilizing siRNA-mediated silencing of the gene expression. Such genetic depletion assays have been repeatedly combined with proteome analysis to identify and characterize potential endogenous protease substrate polypeptides in mammalian cells. Despite the severe and obvious phenotype of Lon mutants, the amount of information on its proteolysis properties in mammalian cells is still fairly limited. A study based on a doxycyclin-induced siRNA-mediated depletion of Lon combined with a 2D-PAGE-based analysis of the mitochondrial proteome observed a multitude of negative effects on mitochondrial protein composition and functions [29]. The effects also included an upregulation of autophagy flux correlating with a general cellular damage, but unfortunately no specific mitophagy assays were performed in this study. However, the main problem of this study was that no controls for an effect of the doxycyclin treatment alone were performed. The antibiotic doxycyclin is a known inhibitor of mitochondrial translation [30] so it cannot be excluded that all observed mitochondrial deficiencies were based on this effect and may have no connection to the Lon depletion as such. A recent study used heterozygote genetic knockout *LONP1*^+/−^ MEF cells, exhibiting a substantially reduced level of Lon, and performed an extensive quantitative mass spectroscopy-based characterization of the cellular proteome [31]. Although a multitude of technically significant changes in protein levels have been detected in this study, most alterations, in particular concerning mitochondrial proteins, remained relatively minor (a few percent change) so that any conclusions about biological significance, reflecting important functional alterations, remained questionable. It is rather likely that moderate alterations of protein levels in genetic knockdown or depletion experiments reflect long-term adaptation processes where biogenesis processes have been fine-tuned to maintain the overall functionality of a cellular system like mitochondria. A direct conclusion on potential specific protease substrates from these experiments is therefore practically impossible without a confirmation by direct and dedicated proteolysis assays.

Functionally, it is been well conceivable that also the Lon protease in mammalian mitochondria cooperates with other chaperone enzymes to deal with damaged polypeptides. The likely candidates for chaperone co-operatively with Lon are the matrix chaperones Hsp60 and mtHsp70. Indeed, a proteomic screen for Lon-interacting proteins identified over 70 potential interacting polypeptides, prominently including the two chaperones [32]. However, only seven of these potential integrators were actually localized inside mitochondria, so it must be concluded that most of the found associations were likely nonspecific. In addition, the overall approach of the interaction analysis involved an overexpression of Lon as well as the chaperone proteins as GST-tagged fusion proteins. Under these conditions, it is highly probable that the detected interactions were artificial due to recognition of the respective protein constructs as simple chaperones substrate proteins. It has to be noted that direct physical interactions—in form of a mechanistic cooperation during the enzymatic process—between molecular chaperones and the Lon proteases from different systems have not been detected so far.

### 2.2. Protection against Oxidative Stress

Due to their oxidative metabolism, mitochondria represent the main sources of cellular reactive oxygen species (ROS). ROS can react nonspecifically with amino acid side chains in proteins, covalently modify them, and thereby cause irreversible damage, resulting in the destruction of protein function or structure. One of the first identified endogenous substrate proteins of the Lon protease in mammalian cells was the Krebs cycle enzyme aconitase [33]. This enzyme contains an Fe/S cluster and is therefore very susceptible to oxidative modifications. Although the actual degradation rates remained fairly low—only a few percent of ROS-damaged aconitase were degraded even after longer incubation periods, this work set that hallmark of Lon as a major PQC component involved in preventing the accumulation of damaged proteins under oxidative stress.

Subsequently, a characterization of the yeast mitochondrial proteome under oxidative stress identified a multitude of proteins that were degraded after a treatment with ROS-inducing chemicals [34,35]. These studies also demonstrated that Pim1 is generally responsible for removing ROS-damaged soluble polypeptides and that Pim1-mediated proteolysis increased the cellular resistance to external oxidative stress. As ROS-induced chemical modifications of proteins are generally covalent and cannot be repaired, a proteolytic removal of damaged polypeptides is the only way of preventing further mitochondrial defects. Pim1 in yeast and its relative Lon in mammalian cells therefore seem to be the main PQC component that is involved in the maintenance of the mitochondrial soluble proteome under certain stress conditions. However, note that it does not have a direct protective function against stress as such, but rather mitigates the consequences of certain stress processes on the general functionality of mitochondria. 

A recent work claimed that in “damaged” mitochondria, characterized by a low inner membrane potential (∆ψ), Lon (in concert with ClpP) might even have a direct protective role against oxidative stress by degrading subunits of the respiratory complex I, which is a major source of ROS under these conditions [36]. Although it is conceivable that at least peripheral subunits of complex I may be substrates of the matrix proteases, only very small effects on the amounts of complex I components were observed after knockdown experiments of both proteases at low ∆ψ. In particular, the report did not include a direct analysis of ROS levels under these conditions so a direct causal relationship between complex I degradation by Lon and a decreased ROS production was not demonstrated.

### 2.3. Prevention of Aggregation

Protein aggregation represents a major biochemical pathology in many important human diseases, in particular, neurodegenerative diseases. However, note that none of the so far identified human pathologies exhibited a prominent accumulation of protein aggregates inside mitochondria. The only exception might be amyotrophic lateral sclerosis (ALS) that affects a mitochondrial protein, superoxide dismutase 1 (Sod1)—although Sod1 has a dual localization in the cytosol and in the intermembrane space [37] and the aggregated material likely also accumulates in the cytosol. It is unclear, why mitochondria do not prominently exhibit aggregate pathologies. Interestingly, artificial aggregation constructs based on poly-glutamine fusion proteins failed to produce aggregates when targeted to mammalian mitochondria [38]. Recently, using different forms of aggregation reporter proteins, a specific process of dealing with aggregated polypeptides in the mitochondrial matrix has been demonstrated, a sequestration of aggregates into inert vesicular-like sub-mitochondrial compartments (IMiQs) that allows to prevent further damage to the organelle [39], potentially contributing to the apparent resistance of mitochondria against protein aggregation. 

It is important to consider that the specific biogenesis process of mitochondrial proteins usually requires unfolding/refolding reactions as well as assembly processes of polypeptides expressed from two different genetic sources, all performed in an oxidatively challenging environment [40]. It is therefore likely that mitochondria have to deal with a high degree of protein conformation problems, which carry an intrinsic likelihood of aggregate generation [41]. Already in the initial publication it was observed in EM pictures that *pim1∆* yeast cells exhibited an accumulation of electron-dense particles in the mitochondrial matrix. This indicated an involvement of Pim1 in the removal of surplus or defective aggregation-prone protein material. In isolated yeast mitochondria, import of artificial destabilized reporter proteins resulted in protein aggregation that is dealt with by the internal PQC system in a specific fashion. The mitochondrial ClpB-homolog chaperone, Hsp78 has been shown to be responsible for the active resolubilization of aggregated polypeptides in the matrix compartment [42]. The now resolubilized reporter proteins where then directly degraded by the Pim1 protease, most likely to prevent the re-aggregation of these unfolded and instable polypeptides generated by the ClpB complex. In a similar fashion, Pim1 was also shown to reduce the accumulation of endogenous protein aggregates in heat-treated mitochondria together with other components of the PQC system [43]. Interestingly, oxidative stress as such was not observed as a major cause for protein aggregation, indicating that an oxidative modification of amino acid side chain may not cause widespread structural changes leading to complete denaturation. As noted above, the ROS-induced protein damage may also be efficiently dealt with by the mitochondrial proteases, reducing the risk of aggregate formation. Also, in mammalian mitochondria, it was shown that elevated temperatures generated protein aggregation, although many proteins were remarkably resilient against this type of stress conditions [44]. Interestingly, one of the most aggregation-prone mitochondrial proteins was the translation factor Tu (encoded by *TUFM*), suggesting that a shutdown of mitochondrial translation under stress conditions is a major protective mechanism to prevent accumulation of damaged proteins.

A recent article described a potential additional way how mitochondrial Lon might deal with aggregated polypeptides in yeast cells. Here, it was claimed that aggregates formed in the cytosol would be taken up by the mitochondrial import machinery of the outer and inner membranes, transported into the matrix compartment, and then degraded by the protease Pim1 [45]. As such, this claim contradicts several well-established properties of mitochondrial import [46] as well as Lon-mediated proteolysis. (i) A multitude of experiments in vitro and in vivo—most of them cited in this review—clearly demonstrated that mitochondrial Lon is not able to degrade already aggregated polypeptides. (ii) In addition, due to the small dimensions of the mitochondrial import pores, polypeptides can only pass the membranes in a completely unfolded conformation, essentially preventing the uptake of protein aggregates. To reach the mitochondrial matrix for degradation, the aggregated polypeptides would need to be resolubilized first. As such, a reaction like this might be possible—driven by the activity of the cytosolic chaperone Hsp104 [47]. However, the authors did not directly demonstrate that their aggregated reporter protein were actually disaggregated by Hsp104. (iii) Even if this disaggregation reaction would take place, it would be unlikely, even impossible, for the used reporter proteins to be taken up by mitochondria because they lack any targeting signal that is normally required for mitochondrial import. Indeed, an uptake of the reporter proteins into the matrix compartment was again not directly shown by the authors in biochemical assays. This criticism also applies to the observation that isolated mitochondria with a mutation of the core inner membrane translocase pore, Tim23, showed a delayed degradation of the aggregated reporter in vitro assuming that the import (and subsequent degradation by Pim1) would be inhibited. In this context, the actual uptake was not directly shown as well as the disaggregation, in particular, as in the in vitro system, the cytosolic chaperone Hsp104 would not be present anyway. In addition, the main protein import assays utilized in the article represent a rather artificial in vivo set-up based on split-GFP fusion proteins—the major part of the GFP protein as a reporter for localization inside mitochondria and the minor part fused to a heat-sensitive aggregation-prone polypeptide in the cytosol. The reasoning was that if a fluorescent GFP signal was observed in the microscope, the aggregate reporter was getting into contact with the mitochondrial reporter in the matrix compartment. However, the used conditions to induce aggregation (and the claimed mitochondrial uptake of the aggregate), in particular, heat shock and depletion of the inner mitochondrial membrane, typically abolish mitochondrial import per se. Therefore, a perfectly conceivable alternative explanation for an observation of the split-GFP signal is that the supposedly mitochondrial reporter GFP-part was actually not imported any more into mitochondria under these conditions and contacts his aggregate counterpart simply in the cytosol at the mitochondrial surface—with the aggregated polypeptides never actually entering the mitochondria. Unfortunately, the microscopic visualization used does not allow distinguishing between these alternatives. Taken together, the uptake and Pim1-dependent degradation of cytosolic aggregates represents an intriguing hypothesis, but definitely requires a much more detailed experimental conformation to be generally acceptable. 

### 2.4. Role in Gene Expression

Early observations indicated Already that the Lon protease at least indirectly affected mitochondrial gene expression. In the yeast model system, a deletion of the *PIM1* gene resulted in reduction of the biosynthesis of specific mitochondria-encoded polypeptides that had genes containing intron sequences [48]. As chambered proteases also typically exhibit chaperone activity that can be independent of the proteolytic function, the question arose if this was also the case for Lon-type proteases. Indeed, overproduction of a proteolytically inactive mutant form of Pim1 in yeast was able to rescue the growth defects of a genetic deletion of the inner membrane m-AAA protease, hinting at additional functions besides proteolysis [49]. However, instead of having an intrinsic chaperone function, as was postulated, the effects of both proteases on respiratory complex assembly is rather indirect and most likely related to their involvement in the expression of intron-containing mitochondrial genes in yeast [48,50]. However, as the underlying biochemical mechanism of this effect was not determined, the existence of an intrinsic chaperone activity of Lon proteases remains unclear so far. As the mitochondrial genomes in mammalian organisms do not contain introns, a generalization of this observation seems to be questionable. However, a genetic depletion study of Lon in Drosophila flies found a moderate decrease of mitochondrial functions, including some reduction of respiration (but only complex I- and IV-mediated) and some decrease of mitochondrial translation. Both observations correlate well because a decrease in the biogenesis of mitochondrially encoded proteins (including folding and/or assembly into active enzyme complexes) will directly affect the oxidative phosphorylation efficiency [51,52].

In case of the Lon proteases, it has to be considered that they indeed fulfill a secondary role that may neither be directly connected with a function as a protease nor as a chaperone. Lon proteases in bacteria and mitochondria have been shown to be DNA-binding proteins with a potential involvement in mitochondrial DNA replication and/or gene expression [53]. Although the molecular details of the role in mitochondrial gene expression still need to be worked out, the DNA interaction properties of Lon might help to link the metabolic state of mitochondria—via ATP levels—and even the proteostasis fitness—via the presence of substrate proteins—to mitochondrial DNA metabolism [54]. Human mitochondrial transcription factor A (mtTFA, encoded by the gene *TFAM*) is a central factor involved in essentially all process of mitochondrial genome replication and gene expression [55]. Its function ensures the maintenance of the mtDNA under normal or stress conditions. Due to its importance and its localization, a connection between Lon—as a DNA binding protein in mitochondria—and mtTFA was assessed, focusing on the question if mtTFA might be a substrate of the Lon protease. It was speculated that Lon might thereby exert a regulatory effect on mitochondrial gene expression. Indeed, in Drosophila cells, Lon overexpression or siRNA knockdown experiments showed at least a moderate increase or reduction in mtTFA and mitochondrial DNA levels, respectively [56]. However, this observation remains a correlation as the authors did not perform a direct degradation assay in intact cells or at least in intact mitochondria. Alternatively, a slight adaptation of mtTFA levels under the used conditions would be conceivable as well without the involvement of an actual proteolysis reaction. Another prominently published paper claimed that mtTFA DNA-binding is regulated by phosphorylation and that DNA-free mtTFA would be degraded by the Lon protease [57]. However, these conclusions also have to be critically assessed as the actual degradation reactions have only been performed in a complete in vitro setup with purified proteins. Under in vivo conditions, only artificially expressed mutated versions of mtTFA that contained to types of amino acid exchanges (supposedly either interfering with DNA-binding or phosphorylation) were shown to be degraded. However, the authors neglected to assess the folding state of the protein constructs, so it cannot be excluded that the mutated mtTFA protein is structurally destabilized and therefore degraded just as any other misfolded PQC substrate protein. Note that an effect of mtTFA phosphorylation, claimed by the authors to be a “degradation signal” for the Lon protease, could only be shown when the respective kinase enzyme was artificially targeted to the mitochondrial matrix by fusing it with a mitochondrial targeting sequence. Under these artificial conditions, any conclusion on an effect of phosphorylation on recognition and degradation of mtTFA by Lon is essentially impossible.

## 3. Involvement in Metazoan Cellular Stress Protection

Mitochondria and their eukaryotic host cell have become interdependent biological system whose survival depends on a fine-tune balance of a multitude of functional and physical interactions. External stress conditions can perturb this balance and cause cellular dysfunction and disease. Mitochondria can react at different levels to such perturbations [7]: (i) the molecular level, at which the PQC system prevents damage to proteins; (ii) the organellar level, at which damaged mitochondria are specifically removed; and (iii) the cellular level, where terminally damaged mitochondria signal cellular death (apoptosis). As the Lon protease represents an important basic activity in this protective system, many pathological processes, e.g., oxidative stress protection, aging and cancer are potentially affected by the activity of Lon [58,59,60].

### 3.1. Stress Induced Upregulation of Lon

Components of the cellular PQC machineries have been recognized as proteins that exhibit higher levels under stress conditions, leading to an adaptation of the correlated polypeptide stabilization or repair capacities, summarized as UPR process. A typical example is the temperature-induced upregulation of chaperone proteins, also known as heat shock response [61]. Note that the typical mitochondrial chaperones from the Hsp60 and Hsp70 families do not exhibit this strong heat shock response in comparison to some of their cytosolic or bacterial counterparts [62]. At least in yeast cells, the overall reactivity of the mitochondrial proteome to temperature-induce stress reaction seems to be rather low [24,35]. This seems to be also the case for components for the mitochondrial proteases in general. Interestingly, the Lon protease does not seem to be temperature-inducible but a moderate upregulation of its levels were typically observed under oxidative stress conditions, both in yeast [35] as well as in mammalian cells [63]. This upregulation nicely correlated with its important role in the removal of ROS-damaged polypeptides. As a certain twist to the story with so far unknown relevance, this (slight) upregulation—at least in Drosophila flies—was found to be sex- and age-dependent as well as differentially influenced by the type of ROS treatment [64]. Another specialized case was demonstrated in steroid hormone producing cells that are dependent on the cholesterol transporter protein StAR that resides in the inner membrane [65]. Due to the high amounts of StAR protein, some polypeptides seem to be mis-localized into the matrix compartment where they require Lon for their degradation. In response these cells exhibited a transcriptional activation of the Lon protease gene to adapt to this high prevalence of a specific substrate protein.

An interesting approach was taken by combining a genetic depletion with a co-expression of a protease-defective Lon protease to amplify potential phenotypic effects. In this study aggregation of cellular polypeptides was analyzed and—although the absolute amounts remained unclear—several mitochondrial preproteins were found in aggregated form, potentially reflecting a mitochondrial protein import and/or processing deficiency [66]. Although a preprotein import defect would be consistent with the known indirect effects of Lon defects on mitochondrial energetics, the data for a claimed physical interaction between Lon and the mitochondrial preprotein processing protease (MPP) were not convincing. The used interaction assays only showed negligible amounts of the Lon protease binding to MPP that were technically not distinguishable from background levels. Interestingly, this study did not observe a significant upregulation of mitochondrial chaperones under Lon depletion conditions, even on the mRNA level, which would be indicative of a potential mtUPR. Instead, the authors claim that loss of Lon elicits a so-called integrated stress response (ISR), essentially representing a cell-wide prosurvival, homeostatic gene expression program [67]. As this ISR was observed only after several days of Lon depletion and did not entirely correlate with the reduced Lon levels, it needs to be considered that the observed ISR activation rather was cause by a long-term accumulation of nonspecific cellular damage that is not directly connected to Lon or even mitochondrial activity.

### 3.2. Role during ER Stress

Organelle contact sites have been described as important cellular hubs for specific processes in the recent years [68]. Here, the functional and physical interaction between mitochondria and the ER, summarized under the expression mitochondria-ER contact sites (MiERCs) has become a prominent example, with functions reaching from the regulation of Ca^2+^ ion concentration, phospholipid biosynthesis as well as regulation of mitophagy or mitochondrial dynamics. Interestingly, some recent articles (see below) also claimed a direct involvement of the Lon protease in this specific inter-organellar communication system—although, as a protein complex localized in the mitochondrial matrix, a direct physical interaction of Lon with protein components of ER membranes appears unlikely if not impossible.

A direct relation between ER stress reactions and a concomitant response of the mitochondrial PQC system was claimed on results obtained by a treatment of cultured cells with different toxins affecting ER secretory protein maturation and transport (tunicamycin and brefeldin A) or ER calcium metabolism (thapsigargin) [69]. Under these conditions, minor increases in Lon and the matrix chaperone mtHsp70 levels were observed in contrast to a strong elevation of the ER chaperone BiP (encoded by *HSPA5*; also named Grp78), as expected. In mitochondria, also some, but not all, COX subunits were found reduced after these treatments, suggesting an increased degradation by Lon. However, a direct assay proving that Lon was actually responsible for this reduction was not performed. It is more likely that the chemical treatments caused pleiotrophic defects in the overall cellular fitness, including a reduction of cytosolic translation efficiency. This would of course indirectly affect also mitochondrial protein homeostasis and the composition or activity of respiratory complexes. As the authors did not provide any mechanistic explanation for the claimed “transmission” of ER stress to the mitochondria, the conclusions drawn are unreliable, at least unless independent conformation of this hypothesis has been provided. Similarly, a careful evaluation has to be undertaken of a recent work again claiming Lon protease as a mediator between drug-induced mitochondrial dysfunction and ER stress [70]. The chemical treatments with ER stressors only resulted in very small effects on Lon protease levels—quite in contrast to realistic targets like endogenous ER chaperones. In addition, the claimed direct association of Lon with mitochondria-ER contact sites was performed using essentially two assays, one based on confocal microscopy and the second on the biochemical preparation of mitochondria-associated membranes (MAM). Both assays exhibit serious drawbacks concerning a characterization of MiERCs so that any conclusions drawn from them have to be regarded with extreme caution. The resolution (in all three dimensions) of confocal optical microscopy (minimally at 200 nm even in the best cases) is typically unsuited to resolve MiERCs as they represent structures with a membrane distance of at most 30 to 50 nm by definition [71]. The MAM preparation, which is based on the purification of membrane structures from crude mitochondria [72], is problematic as it typically does not allow a distinction between a simple contamination by ER membrane proteins and the presence of real MiERCs structures. This problem is even exacerbated by using an equal protein load of all cell fractions in the analysis [70], which strongly enhances the signals of minor contaminating proteins. In this context it has to be noted that the proteins calnexin and porin, claimed by the authors as “markers” for MiERCs both belong to the most abundant components of the ER and the mitochondrial outer membrane, respectively [73]. In addition to these problems in technical quality of the performed assays, the quantitative differences between many drug treatments and the controls are rather small, raising the question if these observations have a biological significance. Therefore, a direct role of the Lon protease in the functional cooperation between mitochondria and the ER remains unclear so far. Unfortunately, most of these experiments have been performed using rather severe toxin treatments to manipulate either mitochondrial or ER processes, as the details of the mechanism of action and also potential side effects or nonspecific consequences of these treatments are not entirely defined.

### 3.3. Role in Aging

The overall efficiency of cellular proteostasis reactions have been identified as an important contributing factor to aging processes [74]. In general, it is thought that a reduced activity of cellular damage control enzymes contributes to premature aging and, vice versa, an upregulated activity of PQC systems would contribute to lifespan extension [74,75]. As aging processes also correlate with energy metabolism efficiency and cellular ROS concentrations as a major factor in generation of molecular damage, the role of mitochondria in this context is obvious. Therefore, also the Lon protease as a prominent enzyme in ROS-related mitochondrial PQC reactions had been implicated in the aging process. Indeed, muscle samples from aged or ROS-stressed animals exhibited lower levels of Lon, concomitant with an increase in the amount of ROS-modified proteins [76]. This correlates also with earlier findings of age-affected gene expression. Here, levels of Lon were found strongly reduced [77]. Related with this observation, a deletion of the Pim1 protease in yeast cells resulted in accelerated aging [78]. Also in mammals, it was observed that the stress adaptability via Lon upregulation is decreasing in older cells, a phenomenon that obviously will contribute to an accelerated aging process [79]. As a confirmation of the role of mitochondria for cellular aging, at least in fungi, an enhanced mitochondrial PQC efficiency contributed to an increased fitness and life span [80]. In addition, a longevity model system in mice showed that an increase in the level of mitochondrial PQC components, here mtHsp60 and Lon were analyzed, correlated with an increased life span. On the other hand, Lon knockdown experiments in Drosophila flies confirmed an overall shortened lifespan, and a reduced respiration efficiency that was even more pronounced in older flies [51]. Taken together, these observations generally support the important role of mitochondrial fitness on cellular aging processes. However, Lon as such is very likely not a distinct “aging factor”, but influences aging indirectly by its function as a member of the mitochondrial PQC system that is required for maintenance of mitochondrial function.

### 3.4. Role during Cancer

Tumor development and progression on a cellular level is driven by typical genetical alterations leading to a de-differentiation and uncontrolled multiplication of affected cells. In particular, in the early stages of this tumor transformation, growth conditions inside a tumor are characterized by a micro-environment with low-oxygen conditions [81]. It has been long known that the transformation of normal cells into tumor cells involves a reprogramming of the energy metabolism away from aerobic respiration, which is mainly mitochondria-based, towards glycolytic energy production, which requires less oxygen—a phenomenon called “Warburg effect” [82]. As this metabolic reprogramming severely affects mitochondrial functions, recent studies addressed the role of the Lon protease in tumor development [60]. It was observed that cancer cells exhibited a moderately increased level of Lon protease [28,83]. On the other hand, a reduction of Lon seemed to protect to a certain degree against tumor transformation. Lon overexpression was observed to exert negative effects on the composition of the respiratory complex I, resulting in an increased ROS production. This in turn elicited growth-promoting signaling processes, which would support an indirect tumor-promoting effect of Lon [84]. Indeed, a retrospective study of cancer patients revealed that higher Lon levels correlated with more aggressive tumor growth and lower survival rates [85].

Cellular adaptation processes under hypoxic conditions mainly rely on the action of the hypoxia-induced transcription factor (HIF) [86]. HIF was shown to regulate the differential expression of certain subunits of the cytochrome c oxidase (COX; complex IV of the respiratory chain) that also is thought to represent at least an aspect of the metabolic reprogramming process in adaptation to hypoxic conditions typical for a tumor microenvironment [87]. Interestingly, Lon was also shown to be upregulated by HIF under these conditions—the promotor of the Lon gene exhibits several HIF binding sites. Such COX remodeling under low oxygen conditions was found to be reduced in Lon depletion mutants. In this context, it has to be noted that Lon might be relevant for the survival of cells not only in tumors but also after ischemic injuries. Similar to other stress situations, Lon was found upregulated in hypoxic cardiomyocytes [88]. Its amounts also affected apoptosis rates and ROS production correlating with its function in mitochondrial proteostasis. In this study, it was also claimed that the Lon protease is also degrading specific subunits of the COX complex under conditions of hypoxia and myocardial ischemia, reinforcing a potential protective role of Lon under pathological conditions [89]. However, as Lon classically is responsible for the degradation of soluble substrate proteins and the inner membrane-integrated polypeptides are mainly substrates of the membrane AAA-proteases, the mechanistic role of Lon in the remodeling of membrane integrated respiratory complexes of these effects on the is still undefined. Although a strong reduction of nuclear encoded COX subunits was observed in hypoxic mitochondria, Lon levels showed only a minor increase at the same time. In addition, these studies again did not directly demonstrate that Lon was actually responsible for the degradation reaction in vivo. Although, it is likely that Lon defects lead to a generalized mitochondrial dysfunction that decrease cellular fitness, eventually even leading also to an increase in apoptosis rates [52] and that an overexpression of Lon might be supportive for metabolic reprogramming in a transformation into cancer cells, a better definition of the involvement of Lon in such pathological conditions definitely requires more dedicated studies. 

## 4. Summary and Outlook

**Figure 2:**
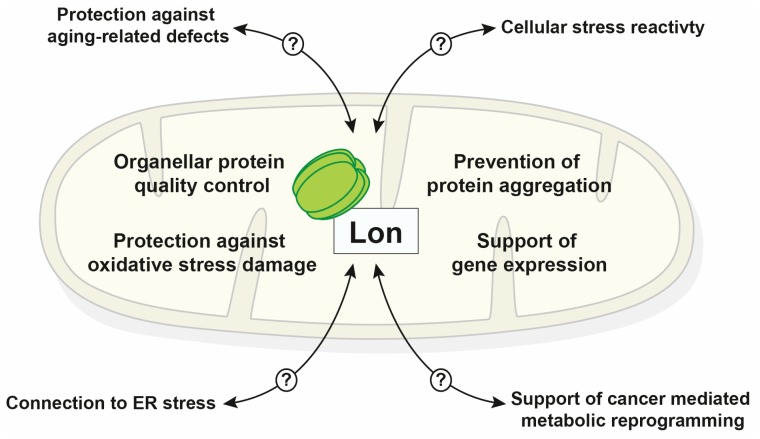
Schematic summary of known Lon functions inside mitochondria and cellular processes that exhibit a direct or indirect involvement of the protease (endoplasmic reticulum (ER)).

Past experiments have firmly established the Lon protease as a central member of the mitochondrial PQC system. Recent studies also indicate a potentially wide array of cellular functions that exhibit at least an indirect involvement of Lon (Figure 2). Its basic function is the removal of damaged polypeptides to counteract accumulation of misfolded proteins and aggregation, in particular under oxidative stress conditions. The observed embryonic lethality of a complete deletion mutant in mammalian organisms confirms its importance for embryonic development and cellular survival. Unfortunately, a detailed and complete structural characterization of Lon proteases has not been performed to date so that only indirect conclusions about the molecular basis of its substrate recognition and proteolysis mechanism have been possible so far. Although, a few major substrate proteins have been identified in metazoan cells, a comprehensive picture of Lon-mediated proteolysis is still lacking. Although some quantitative proteomic screens have been performed to identify substrate proteins in mammalian cells, most of them have relied on gene knockdown or silencing experiments. Due to the inherently long time frame of cell growth in presence of low protease levels, it is rather likely that adaptive changes in the proteome instead of actual degradation substrates are picked up using this approach. Instead of a simple characterization of protein levels it is required to perform direct degradation assays to identify real substrate proteins. Provided the proper control experiments are included, it seems more promising to use cell models with a stably reduced Lon levels as model systems to analyze the reactivity of cells with a compromised PQC system to diverse stress or pathological conditions. Due to the potential connection of mitochondrial dysfunction with cellular aging processes, such cell models will be very valuable to decipher molecular mechanisms of aging-related, in particular neurodegenerative, diseases.
